# Phenotypic characterization of indigenous Xhosa goat ecotype in three agro-ecological zones in the eastern cape province, South Africa

**DOI:** 10.1016/j.vas.2025.100512

**Published:** 2025-09-22

**Authors:** Sibulele Praise Ntonga, Oluwakamisi Festus Akinmoladun, Ziyanda Mpetile

**Affiliations:** a Department of Animal and Pasture Science, Faculty of Science and Agriculture, Private Bag X1314, Alice 5700, Eastern Cape South Africa; b Department of Animal and Environmental Biology, Faculty of Science, Adekunle Ajasin University, Akungba Akoko, PMB 001, Nigeria

**Keywords:** Phenotypic characterization, Agro-ecological zones, Xhosa goats, Breed characterization, Genetic improvement, Body indices

## Abstract

This study characterized the phenotypic attributes of indigenous Xhosa goats across three agro-ecological zones and developed predictive models for estimating body weight. A total of 450 Xhosa goats were sampled using a stratified random approach based on sex, age, and agro-ecological region. Morphometric traits, including body weight (BW), heart girth (HG), body length (BL), wither height (WH), and body depth (RD), were recorded. Data were analysed using the General Linear Model (GLM) of SPSS (v.20) to assess the effects of zones, sex, and age on body traits, while Pearson’s correlation and stepwise regression identified the best predictors of body weight. Results showed significant (p < 0.05) variations in body measurements across zones, with Savanna goats exhibiting superior traits. Males had significantly (p < 0.05) higher BW and body dimensions than females and castrates. Correlation analysis revealed HG (r=0.80), BL (r=0.84), and RD (r=0.82) as the strongest predictors of BW in males, while BL (r=0.66), HG (r=0.65), WH (r=0.62), and RD (r=0.61) were best for females. Stepwise regression identified HG and BL as the best predictors for males, and HG, BL, and SH for females. The predictive models (R² = 0.74–0.85) provide a practical tool for estimating body weight in field conditions. These findings provide practical tools for field-based weight estimation and highlight the importance of conserving the phenotypic diversity of Xhosa goats for sustainable breeding and genetic improvement programs in South Africa.

## Introduction

South Africa is home to a rich diversity of indigenous goat breeds (DAFF, 2012), most of which are non-descript and managed under traditional systems by rural and communal farmers (Bester et al., 2009; du Toit et al., 2014). These indigenous goats represent a valuable genetic resource for smallholder farming communities, having undergone natural selection and adaptation to diverse production systems over time. Their ability to thrive in harsh environmental conditions is attributed to their resilience and efficient utilization of marginal resources (Akinmoladun et al., 2019, 2020). Despite their adaptability, these local goat populations exhibit considerable phenotypic and possibly genotypic heterogeneity, an untapped potential that could be leveraged through research to enhance their productivity and conservation (Akintunde et al., 2024).

Although studies investigating the influence of demographic factors, traditional management practices, and evolutionary trends on indigenous goats in South Africa have gained traction in recent years, research on these animals remains limited compared to other livestock species. A key factor contributing to this research gap is the perception that indigenous goats are primarily livestock for resource-poor, marginalized communities. Consequently, funding and research efforts have predominantly focused on cattle, which are traditionally regarded as the main source of meat (Schiere and Kater, 2001; Sebei et al., 2004). Among the indigenous goat ecotypes, the Xhosa goat—one of the three ecotypes of the non-descript indigenous veld goat—is widely distributed in the rural areas of the Eastern Cape Province. The other ecotypes include the Nguni and the Northern Cape Speckled goats (Morrison, 2007; DAFF, 2012). These ecotypes not only constitute the majority of the goat population in communal farming systems but also serve as key genetic resources for the development of commercial meat-type goats (Campbell, 2003; Bester et al., 2009).

One of the major challenges in characterizing indigenous goat populations in South Africa is the inconsistency in nomenclature. The classification system used often groups goats from different zoness under a single designation, failing to accurately reflect their genetic diversity (Mdladla et al., 2017). Additionally, uncontrolled breeding and poorly planned crossbreeding practices by rural farmers pose a significant threat to the genetic integrity of these populations (Gizaw et al., 2011; Celik, 2019). While intended to improve productivity, such practices risk genetic dilution, breed extinction, and the loss of vital traits such as disease resistance and resilience (Khan, 2018). Given the limited research and the inherent phenotypic variability among indigenous goats, further studies are needed to assess and document their genetic resources to support conservation and sustainable utilization efforts.

Comprehensive breed characterization is essential for effective conservation and sustainable use, as it entails both qualitative and quantitative description of the animals and their production systems. Phenotypic characterization, based on morphological traits, provides critical insights into genetic diversity and adaptive traits, which are essential for developing effective breeding strategies and improving productivity (Nguluma et al., 2016). Furthermore, phenotypic variation reflects the adaptability of goats to different agro-ecological zones, highlighting their potential for sustainable production under diverse conditions (Yakubu et al., 2010; Haga et al., 2012). Body measurement, in addition to weight estimate, describes the individual or population, unlike conventional weighing and grading on a small scale (Salako, 2006). Body dimensions have been used to indicate breed, origin and relationship through the medium of head measurements.

Despite some efforts to characterize the Xhosa goat, morphometric variations across different agro-ecological zones remain largely unexplored. The three study zones were intentionally chosen to capture an eco-climatic gradient expected to influence phenotypic expression in the Xhosa goat ecotype. The Savanna zone provides mixed woody–grassy vegetation and moderate rainfall that supports diverse browse; the Nama Karoo is semi-arid with sparse shrubs and episodic forage availability; and the Grassland zone is cooler, generally higher in altitude, and dominated by seasonal grasses. These contrasts are relevant because forage quality and quantity, thermal load, and altitude collectively modulate growth trajectories and body conformation in small ruminants. We therefore hypothesized that (i) goats from Savanna would exhibit larger linear measurements due to better year-round forage; (ii) goats from Nama Karoo would show relatively compact morphometrics associated with heat and drought constraints; and (iii) goats from Grassland would present greater skeletal heights and lengths consistent with cooler temperatures and seasonal nutrition. Selecting these zones also reflects where Xhosa goats are widely kept by smallholders under comparable extensive systems, ensuring that ecological effects on phenotype can be examined under standardized measurement protocols.

This study, therefore, aimed to characterize the phenotypic attributes of the indigenous Xhosa goat ecotype across three distinct agro-ecological zones in the Eastern Cape Province of South Africa, while also developing predictive models for estimating body weight from linear body measurements. These contributions fill an important knowledge gap and provide practical tools for both conservation and genetic improvement programs.

## Materials and methods

### Ethical clearance

This study received approval for the use of animals based on the guidelines approved by the Animal Research Ethics Committee of the Faculty of Science and Agriculture, University of Fort Hare (Ref: MPE03SNTO01/23/A).

### Study site description and goats’ management

**Fig. 1:**
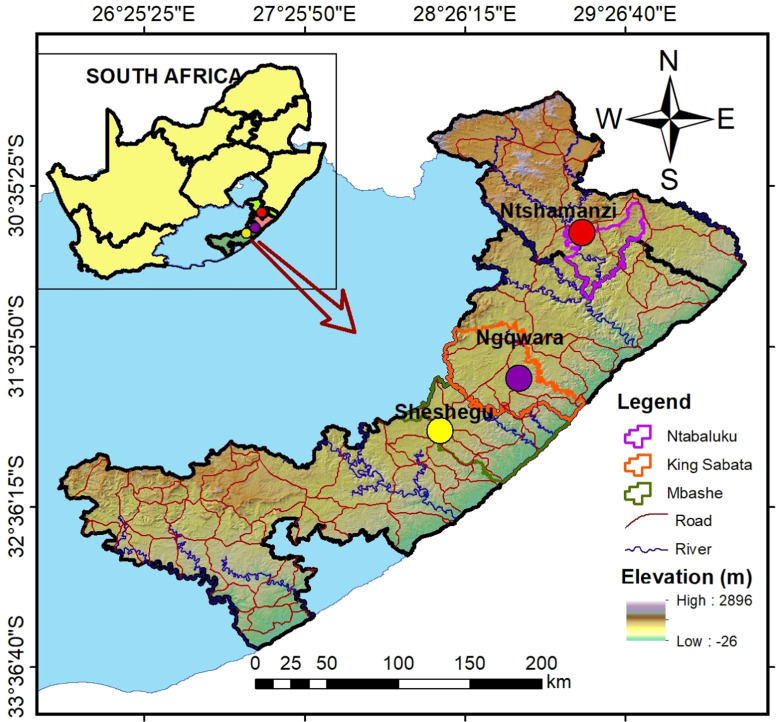
Location map of the sampling zones.

The study was conducted in the Eastern Cape Province of South Africa, focusing on three major agro-ecological zones, Savanna, Grassland, and Nama Karoo, as classified by Ndhleve et al. (2021). A multistage sampling approach was used to ensure representative coverage of Xhosa goat populations. In the first stage, one representative location was selected from each zone, namely Sheshegu (Savanna), Ngqwara (Nama Karoo), and Ntshamanzi (Grassland). These locations were chosen because they host substantial populations of Xhosa goats and present contrasting ecological conditions that could influence phenotypic traits. The location map is shown in Fig. 1.

The three zones differ markedly in climate, vegetation, and topography. Sheshegu (Savanna) receives moderate rainfall (450–600 mm annually) and is dominated by Bisho Thornveld vegetation consisting of thorny shrubs and scattered grasses adapted to semi-arid conditions. Ngqwara (Nama Karoo) experiences an arid to semi-arid climate with an average annual rainfall of 469.9 mm, characterized by hilly terrain, rolling grasslands, deep gorges, and high diurnal temperature variation with hot days and cold nights. Ntshamanzi (Grassland), by contrast, records the highest annual rainfall (657 mm) and supports extensive grassveld interspersed with patches of coastal forest, providing relatively richer forage resources that may enhance growth and body condition.

These contrasting environmental profiles provided the scientific basis for selecting the three agro-ecological zones. Specifically, they represent distinct combinations of rainfall, temperature, altitude, and vegetation that plausibly drive differences in morphometric traits. Site choice followed four criteria: (1) clear ecological differentiation; (2) established smallholder populations of the Xhosa goat ecotype; (3) feasibility of standardized, early-morning, pre-feeding measurements by the same trained team; and (4) limited recent crossbreeding reported by farmers. Based on these characteristics, we hypothesized that ecological constraints on nutrition and thermoregulation would translate into measurable differences in body weight and linear measurements across zones, after accounting for sex and age (dentition) in the GLM.

Within each selected zone, a stratified random sampling technique was applied to capture a broad spectrum of goat phenotypes. Households keeping Xhosa goats were identified with the assistance of local extension officers and community leaders. From these, goats were proportionally sampled by sex and age class to reflect potential morphological variation. The sample size was determined using Cochran’s formula (95% CI, e = 0.05, p = 0.5), which yielded a minimum requirement of 384 animals. This was inflated by a design effect of 1.17 to accommodate 36 strata (zone × sex × age), giving a target of approximately 450 goats. The chosen sample exceeded both the calculated minimum and the benchmarks set in previous phenotypic characterization studies (e.g., Lorato et al., 2015; Semakula et al., 2010), thereby ensuring adequate statistical power for GLM and regression analyses. Ultimately, 450 goats were measured following standardized protocols to evaluate the influence of agro-ecological variation on phenotypic expression.

### Goats management and data collection

**Fig. 2:**
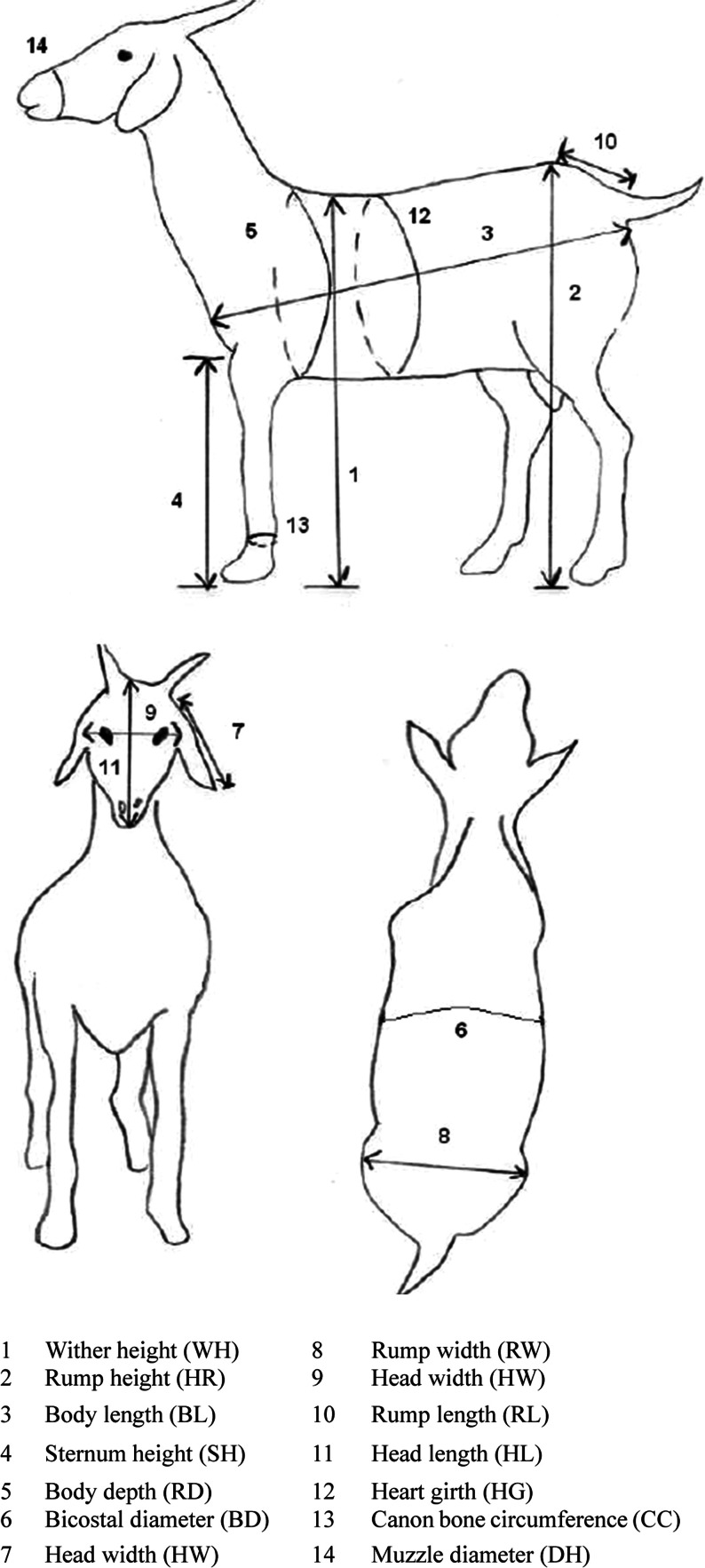
Diagrammatic presentation of the linear body dimensions taken (Source: Chacon et al., 2011).

A total of 450 Xhosa goats were selected for phenotypic characterization. All goats were managed under comparable communal/extensive conditions typical of the Eastern Cape: open grazing without routine concentrate supplementation and no confined housing. To reduce short-term management effects, all measurements were taken in the early morning prior to access to feed or water. A single trained team used the same calibrated tape and protocols across all sites, recording measurements with animals restrained in a natural, upright stance. Each goat was restrained in a natural, upright stance to ensure consistency, and data collection was conducted by the same trained personnel across all study zones. Fig. 2 illustrates the positioning of these body measurements.

Morphological traits were assessed following standardized descriptors established by Morrison (2007) and the Food and Agriculture Organization (FAO, 2012). Quantitative measurements were recorded using a flexible tailor’s measuring tape, calibrated in centimeters, to ensure precision. The parameters measured included body weight (BW), wither height (WH), rump height (RH), body length (BL), sternum height (SH), body depth (RD), bicoastal diameter (BD), ear length (EL), rump width (RW), head width (HW), rump length (RL), head length (HL), heart girth (HG), cannon bone circumference (CC), muzzle diameter (MD), and scrotal circumference (SC) for bucks. Agro-ecological zones, dentition-based age class, and sex were modeled as fixed effects in the GLM to account for ecological and demographic variability.

Each goat was systematically identified based on sex, age, and sampling zones (district). Sex classification included females, intact males (bucks), and castrated males. Age estimation was determined using a combination of farmer-reported information and dental examination, following established guidelines. Goats were categorized into three age groups based on the number of permanent incisor pairs: no permanent incisors (<14 months, 0PPI); one pair of permanent incisors (15–23 months, 1PPI); two pairs of permanent incisors (24–35 months, 2PPI); and three or more pairs of permanent incisors (≥36 months, >3PPI). This classification aligns with standard dentition-based age determination, as goats are born with deciduous (milk) teeth, which are gradually replaced by permanent incisors at specific age intervals.

### Inclusion and exclusion criteria

Goats were eligible if they: (i) were of the indigenous Xhosa-type as reported by owners and consistent with morphological descriptors; (ii) were clinically healthy on visual inspection; (iii) could be reliably assigned to an age class using dentition (0PPI, 1PPI, 2PPI, ≥3PPI); and (iv) had a body condition score (BCS) between 2 and 4 on a 1–5 scale.

Animals were excluded if they presented any of the following: overt illness (e.g., fever, diarrhea, respiratory distress), visible injury/lameness or congenital deformities, severe ecto-/endoparasite burden, extreme BCS (≤1 or ≥5), advanced gestation (final third) or <30 days postpartum (to minimize weight bias), obvious bloat or rumen distension at handling, or if they had received veterinary treatment, dewormers, or vitamin/mineral injections within the preceding.

### Statistical analysis

Before inference, we evaluated GLM assumptions. Normality of residuals was tested with Shapiro–Wilk and inspected via Q–Q plots; homogeneity of variances across factor levels was assessed with Levene’s tests; independence was ensured by sampling distinct animals with no repeated measures; linearity and model fit were examined using residual-versus-fitted plots. Multicollinearity among predictors was screened using variance inflation factors (VIF), with all VIFs < 5. Influential observations were evaluated using Cook’s distance (threshold 4/n); sensitivity analyses excluding flagged points did not change significance patterns or effect directions. Residuals were approximately normal and variances homogeneous; therefore, no transformations were applied. For the stepwise multiple regressions predicting body weight, we repeated residual diagnostics (Shapiro–Wilk, Q–Q, Levene’s on absolute residuals, residual-versus-fitted, VIFs), and the final models met assumptions.

The composition and distribution of the flock were examined through frequency distribution analysis using SPSS software (version 21). To evaluate the least squares means and standard errors of the quantitative linear body measurements, the General Linear Model (GLM) procedure within the same software was applied. Fixed effects included sex, district, and age group of the goats, while body weight and various linear body measurements, excluding scrotal circumference, were treated as dependent variables.

Scrotal circumference was assessed exclusively in intact males, with age and district included as fixed factors in the analysis. Least squares means and their associated standard errors were computed for the fixed effects of sex, age, district, and the interaction between age and sex for each morphological trait.

For body weight and other linear body measurements, excluding scrotal circumference, the least squares mean analysis was conducted separately for males and females using the following statistical model:

$$Y_{ijk}=\mu +N_{i}+D_{j}+G_{k}+{\left(NxD\right)}_{ij}+{\left(NxG\right)}_{ik}+{\left(DxG\right)}_{jk}+e_{ijk}$$

Where *Y_ijk_* = observed body weight or linear measurements; μ = overall mean, *N_i_* = the fixed effect of *ith* age groups (i = 0PPI, 1PPI, 2PPI and ≥ 3PPI), *D_j_* = fixed effect of *jth* zones (j = Savannah, Nama Karoo and Grassland), *G_k_* = the fixed effect of the kth sex (k= male, female, castrate), *(NxD)_ij_* = interaction effect of age group and zones, *(NxG)_ik_* = interaction effect of age group and sex, *(DxG)_jk_* = interaction effect of zones and sex, *e_ijk_* = random residual error, assumed to be independently and normally distributed with mean zero and constant variance.

The model used for the least square mean analysis in males for scrotal circumference was:

$$Y_{ijk}=\mu +N_{i}+M_{j}+{\left(NxM\right)}_{ij}+e_{ijk}.$$

Where *Y_ijk_* = Scrotal circumference; μ = overall mean; *N_i_* = the fixed effect of *ith* age groups (i = 0PPI, 1PPI, 2PPI and ≥ 3PPI), *M_j_* = the fixed effect of *jth* zones (j = Savannah, Nama Karoo and Grassland), (*NxM)ij* = the fixed effect of the interaction of *ith* age group with *jth* of district and *e_ijk_*= random residual error.

Pearson’s correlation coefficients of indigenous Xhosa goat ecotype were estimated between body weight and other linear body measurements (LBMs) within each sex to describe the strength and direction of relationships between the response variable (body weight) and explanatory variables (LBMs). Body weight and other LBMs (e.g. WH, HR, BL, SH) were included for males, whereas SC was excluded when calculating correlation coefficients for female Xhosa goats.

Based on the correlations of body weight with other LBMs, a stepwise regression procedure was then used to regress body weight for each sex in order to determine the best-fit regression equation for the prediction of body weight using LBMs. The best-fit models were selected based on the coefficient of determination (R^2^) and the simplicity of LBM measurements under field conditions. The following models were used for the analysis of multiple linear regressions.

For males:

$$Y_{j}=\beta_{0}+\beta_{1}X_{1}+\beta_{2}X_{2}+\beta_{3}X_{3}+.\ldots +\beta_{n}X_{n}+\beta_{c}C+e_{j}$$

Where: *Y_j_* =the response variable (body weight); *β_0_* = the intercept; *X_1_, X_2_, X_3_ ..…..X_n_* = the explanatory variables (e.g WH, HR, HW); *β_1_, β_2_…β_n_* = the regression coefficients of the variables *X_1_, X_2_…Xn*; C= Castration status (0 for intact, 1 for castrated); *β_c_^=^* Coefficient for castration status (captures the effect of castration on body weight); *e_j_* = Error term.

For female:

$$Y_{j}=\beta_{0}+\beta_{1}X_{1}+\beta_{2}X_{2}+\beta_{3}X_{3}+\ldots +\beta_{n}X_{n}+e_{j}$$

Where: *Y_j_* =the response variable (body weight); *β_0_* = the intercept; *X_1_, X_2_, X_3_.…. Xn* = the explanatory variables (e.g. BL, HG, WH); *β_1_, β_2_…β_n_* = the regression coefficients of the variables *X_1_, X_2_…X_n_; e_j =_* error term. The stepwise regression procedure of SPSS was used to develop prediction equations for body weight (BW) in Xhosa goats.

## Results and discussion

### Flock size and distribution

The flock structure and distribution of the sampled Xhosa goats (450) phenotypically characterized with respect to zones, age and sex are shown in Table 1. The distribution showed variation in age and sex composition within and across agro-ecological zones. In the Nama Karoo, the proportion of older goats (>3PPI) was highest (18.89%), compared with 12.22% in Grassland and only 5.77% in Savanna. Across all zones, females formed the majority of the flocks, reflecting their role as the primary reproductive units in communal systems. This structure suggests that harsher Nama Karoo conditions favor the survival of hardier, older goats, while recruitment and retention of young stock are higher in Savanna and Grassland where forage resources are more favorable. Similar trends were reported in Ethiopian goats, where flock age structure mirrored ecological resilience and management practices (Solomon, 2009). In Uganda, Semakula et al. (2010) also observed female-dominated herds, consistent with the reproductive strategies of smallholder farmers aiming to sustain flock growth. The predominance of females in the current study underscores the importance of reproductive efficiency for household livelihoods. The balanced representation across sex and age groups in this study strengthens the reliability of the subsequent morphometric comparisons.

**Table 1:** Flock structure of indigenous Xhosa ear-lobe goats measured in the surveyed zones of Eastern Cape.

Zones	Fixed effect				Overall	% Composition
	PPI	Females	Males	Castrates		
Savannah	0PPI 1PPI 2PPI 3PP1	42 16 12 13	12 6 3 6	7 6 6 7	61 28 21 26	13.56 6.22 4.67 5.77
Nama Karoo	0PPI 1PPI 2PPI 3PP1	25 6 13 77	13 2 3 4	4 4 4 4	42 12 20 85	9.33 2.67 4.44 18.89
Grassland	0PPI 1PPI 2PPI 3PP1	42 9 20 45	9 3 4 6	7 3 3 4	58 15 27 55	12.89 3.33 6.00 12.22
Total					450	100

### Effect of agroecological zones

The effect of agroecological zones on the quantitative traits of the indigenous Xhosa goats is shown in Table 2. Significant (p < 0.05) differences were observed in most linear body measurements among goats from the three agro-ecological zones, except for body weight, sternum height, rump width, rump length, and head length. Goats in the Savanna region exhibited superior values (P<0.05) for wither height, body length, heart girth, body depth, and cannon bone circumference compared to their Grassland and Nama Karoo counterparts. However, traits such as body weight (BW), sternum height (SH), rump width (RW), rump length (RL), and head length (HL) did not differ significantly among zones.

**Table 2:** Effect of zones on quantitative traits of indigenous Xhosa ear-lobe goats measured in the surveyed zones of Eastern Cape!.

Parameters	Savannah	Nama Karoo	Grassland
BW (kg)	34.84±0.11	34.64±0.14	34.56±0.12
WH (cm)	69.34±0.73^a^	64.39±0.97^b^	61.93±0.86^b^
HR (cm)	72.42±0.85^a^	68.04±1.12^b^	64.573±0.99^b^
BL (cm)	73.95±0.87^a^	71.81±1.15^a^	68.29±1.02^b^
SH (cm)	44.33±0.72	44.13±0.96	42.58±0.85
RD (cm)	78.35±1.16^a^	73.51±1.52^b^	69.08±1.35^b^
BD (cm)	48.89±0.89^a^	44.86±1.17^b^	48.91±1.04^a^
EL (cm)	18.11±0.26^a^	16.72±0.34^b^	18.25±0.30^a^
RW (cm)	17.14±1.16	16.01±1.54	14.59±1.36
HW (cm)	13.13±0.22^b^	14.02±0.29^a^	13.05±0.25^b^
RL (cm)	16.85±0.38	17.47±0.51	16.88±0.45
HL (cm)	23.04±0.57	21.92±0.76	21.31±0.67
HG (cm)	95.51±1.14^a^	87.82±1.89^b^	81.992±1.67^b^
CC (cm)	10.29±0.16^a^	9.33±0.21^b^	9.37±0.19^b^
DH (cm)	23.69±0.29^a^	21.97±0.38^b^	20.95±0.34^b^

I means±SD; BW: body weight; WH: wither height; HR: rump height; BL: body length; SH; sternum height; RD: body depth; BD: bicoastal diameter; EL: ear length; RW: rump width; HW: head width; RL: rump length; HL: head length; HG: hearth girth; CC: cannon bone circumference; DH: muzzle diameter

These results highlight the superior body conformation of goats in Savanna, likely due to moderate rainfall and the availability of browses and grasses in Bisho Thornveld vegetation that provides a more favorable nutritional base (Ndhleve et al., 2021). In contrast, the semi-arid Nama Karoo, with sparse forage and high diurnal temperature variations, may constrain growth, leading to more compact morphotypes. Grassland goats, although living in higher rainfall areas, graze predominantly on seasonal grasses, which may limit year-round nutrient intake compared with the mixed forage base in Savanna.

The ecological influence on morphometrics is consistent with earlier studies. Lorato et al. (2015) reported that indigenous Woyto-Guji goats in Ethiopian highlands with better forage availability were larger than those in arid lowlands. As emphasized by Hagan et al. (2012), phenotypic variability within indigenous goat populations reflects ecological adaptation and provides a foundation for selection and breeding programs tailored to specific production environments. Such morphometric variations across ecological zones highlight the adaptive plasticity of indigenous goats, which is crucial for their survival in diverse environments. Therefore, the phenotypic diversity documented across the three zones in this study underscores the importance of conserving these goats as reservoirs of adaptive genetic traits

### Effect of sex

The effect of sex on the quantitative traits of goats is shown in Table 3. Sex exerted a significant influence (p < 0.05) on most traits. Males had higher body weight and larger dimensions (P<0.05) (BL, WH, HG, CC, BD, RH) compared to females, while castrates occupied intermediate positions. Traits such as head length (HL) were not significantly affected. The superiority of males reflects sexual dimorphism, a well-established phenomenon in livestock. Testosterone and associated hormonal differences promote greater muscle mass and skeletal growth in males (Isaac, 2005). The intermediate values observed in castrates may result from the removal of testosterone’s anabolic effects, leading to body conformations closer to females. Similar patterns have been reported in Ugandan goats (Semakula et al., 2010), Nigerian West African Dwarf goats (Fajemilehin and Salako, 2008) and Woyto-Guji goats in Ethiopia (Lorato et al., 2015).

**Table 3:** Effect of sex on quantitative traits of indigenous Xhosa goats measured in the surveyed zones of Eastern Cape^I^.

Parameter	Females	Males	Castrates
BW (kg)	31.98±0.07^b^	36.08±0.14^a^	35.99±0.13^a^
WH (cm)	61.56±0.50^a^	67.34±1.01^b^	66.76±0.95^b^
HR (cm)	65.34±0.59^a^	70.88±1.16^b^	68.81±1.1^b^
BL (cm)	67.57±0.60^a^	73.60±1.21^b^	72.89±1.14^b^
SH (cm)	41.01±0.50^a^	45.74±1.01^b^	44.30±0.95^b^
RD (cm)	71.73±0.79	74.66±1.60	75.05±1.51
BD (cm)	45.33±0.61^a^	47.67±1.23^ab^	49.65±1.16^b^
EL (cm)	16.83±0.18^a^	17.94±0.36^b^	18.31±0.34^b^
RW (cm)	15.21±0.80	17.05±1.61	15.84±1.52
HW (cm)	12.85±0.15^ac^	14.15±0.30^b^	13.19±0.29^bc^
RL (cm)	16.20±0.26^ac^	17.78±0.53^b^	17.22±0.50^bc^
HL (cm)	21.27±0.40	22.78±0.80	22.22±0.75
HG (cm)	85.08±0.99^a^	89.99±1.99^ab^	90.24±1.89^b^
CC (cm)	9.14±0.11^a^	10.16±0.23^b^	9.71±0.21^b^
DH (cm)	20.99±0.20^a^	22.62±0.40^b^	22.99±0.38^b^

I means±SD; BW: body weight; WH: wither height; HR: rump height; BL: body length; SH; sternum height; RD: body depth; BD: bicoastal diameter; EL: ear length; RW: rump width; HW: head width; RL: rump length; HL: head length; HG: hearth girth; CC: cannon bone circumference; HD: muzzle diameter

The absence of significant sex differences for head length in the present study suggests that certain body parts may have distinct allometric growth patterns less influenced by sex, as previously noted in polar bear skull development (Bechshøft et al., 2008). From a production standpoint, these differences are important for management. While females dominate flocks due to reproductive roles, males offer superior growth traits, making them valuable for meat production. Understanding such differences aids in developing breeding and selection strategies.

### Effect of dentition or age groups

The effect of dentition (eruption of a permanent pair of incisors) or age groups on the linear body morphometrics is shown in Table 4. Age, determined by dentition class, had a highly significant (p < 0.05) effect on all body measurements. Body weight and morphometric traits increased steadily with the eruption of permanent incisors, with the >3PPI group recording the highest values. For example, heart girth and body length showed marked increases between 0PPI and ≥3PPI classes, reflecting growth and development.

**Table 4:** Effect of age on quantitative traits of indigenous Xhosa ear-lobe goats measured in the surveyed zones of Eastern Cape.

Parameters	0PPI	1PPI	2PPI	>3PPI
BW (kg)	30.06±0.11^a^	33.58±0.17^b^	38.96±0.15^c^	43.12±0.12^d^
WH (cm)	55.14±0.79^a^	62.78±1.18^b^	69.59±1.07^c^	73.39±0.85^d^
HR (cm)	57.91±0.91^a^	66.65±1.37^b^	73.12±1.24^c^	75.69±0.98^c^
BL (cm)	60.52±0.94^a^	68.12±1.41^b^	76.47±1.27^c^	80.312±1.01^c^
SH (cm)	36.46±0.78^a^	44.07±1.17^b^	46.71±1.06^b^	47.49±0.84^b^
RD (cm)	61.23±1.24^a^	70.85±1.87^b^	76.95±1.69^b^	85.54±1.34^c^
BD (cm)	38.62±0.96^a^	44.77±1.44^b^	51.16±1.30^c^	55.65±1.03^d^
EL (cm)	16.08±0.28^a^	17.26±0.42^ab^	18.58±0.38^bc^	18.85±0.30^c^
RW (cm)	12.31±1.25^a^	15.45±1.88^ab^	17.18±1.70^ab^	18.72±1.35^b^
HW (cm)	11.22±0.23^a^	12.98±0.03^b^	14.29±0.31^c^	15.09±0.25^c^
RL (cm)	13.78±0.41^a^	16.52±0.62^b^	18.78±0.56^c^	19.17±0.44^c^
HL (cm)	18.59±0.62^a^	22.01±0.93^b^	23.60±0.84^b^	24.17±0.66^b^
HG (cm)	73.59±1.55^a^	84.60±2.32^b^	94.099±2.10^cd^	101.47±1.67^d^
CC (cm)	8.38±0.17^a^	9.46±0.26^b^	10.21±0.24^b^	10.61±0.19^c^
DH (cm)	18.89±0.31^a^	21.41±0.47^b^	23.92±0.42^c^	24.59±0.42^c^

^I^means±SD; BW: body weight; WH: wither height; HR: rump height; BL: body length; SH; sternum height; RD: body depth; BD: bicoastal diameter; EL: ear length; RW: rump width; HW: head width; RL: rump length; HL: head length; HG: hearth girth; CC: cannon bone circumference; HD: muzzle diameter.

PPI: permanent pair of incisors

This trend is consistent with normal growth trajectories in goats, where skeletal and muscular systems continue to mature with age. FAO (2012) emphasizes dentition as a reliable age indicator, particularly under field conditions where birth records are unavailable.

Ethiopian studies also confirmed steady morphometric increases with dentition in goats and sheep (Solomon, 2014; Dereje et al., 2019). The consistency of age-related trends across studies suggests that dentition is a reliable proxy for age classification in indigenous small ruminants, particularly under communal farming systems where birth records are rarely kept. Furthermore, variations in body dimensions within the same age group may reflect environmental constraints such as seasonal feed scarcity, parasite burden, and management practices, all of which can affect growth rates (Semakula et al., 2010)

### Interaction effects

Significant interactions were observed between age × sex, zone × sex, and age × zone for several traits (Table 5). Notably, age × sex interactions were significant for most morphometric measurements, with older males showing the largest increases in BW and body dimensions compared to females of the same dentition class. This reflects the combined influence of maturity and sexual dimorphism on body development (Fajemilehin & Salako, 2008; Lorato et al., 2015). Zone × sex interactions also significantly affected BW and selected morphometrics, indicating that the ecological environment modulates the extent of sexual dimorphism. Comparable findings in Ethiopian goats showed that ecological zones moderated both sex and age effects on body dimensions (Lorato et al., 2015), suggesting that environmental constraints and resource availability amplify phenotypic divergence as animals mature.

**Table 5:** Summary statistics of interaction effects of zones, age and sex on quantitative traits of indigenous Xhosa ear-lobe goats measured in the surveyed zones of Eastern Cape.

Effects	BW	WH	HR	BL	SH	RD	BD	EL	RW	HW	RL	HL	HG	CC	DH
L*xA*	⁎⁎⁎	ns	ns	ns	*	ns	⁎⁎	ns	ns	ns	ns	ns	ns	ns	ns
L*x*S	⁎⁎⁎	⁎⁎	*	*	*	ns	ns	ns	ns	⁎⁎⁎	ns	ns	*	⁎⁎	ns
A*x*S	⁎⁎⁎	⁎⁎⁎	⁎⁎⁎	⁎⁎⁎	⁎⁎⁎	⁎⁎⁎	⁎⁎⁎	⁎⁎	ns	⁎⁎⁎	⁎⁎⁎	*	⁎⁎⁎	⁎⁎	⁎⁎⁎
L*x*A*x*S	⁎⁎⁎	ns	ns	ns	ns	*	ns	ns	ns	ns	⁎⁎⁎	ns	ns	ns	ns

L: zones; a: age; s: sex; ×: interaction.

⁎ :P<0.05

⁎⁎ : P<0.01

⁎⁎⁎ : P<0.001 body weight; WH: wither height; HR: rump height; BL: body length; SH; sternum height; RD: body depth; BD: bicoastal diameter; EL: ear length; RW: rump width; HW: head width; RL: rump length; HL: head length; HG: hearth girth; CC: cannon bone circumference; HD: muzzle diameter.

These results emphasize that morphological traits cannot be fully explained by single factors but instead arise from the combined effects of age, sex, and environment. Importantly, such interactions highlight the necessity of conserving not only the genetic materials of indigenous goats but also the ecological contexts that shape their adaptive traits (Mdladla et al., 2017). The significance of these interactions for BW is particularly instructive because body weight is a key indicator of growth and productivity, directly influencing health, reproductive efficiency, feeding strategies, breeding decisions, and marketing potential. From a genetic improvement and conservation perspective, recognizing that much of the observed diversity stems from interaction effects underscores the importance of safeguarding both the genetic resources themselves and the ecological processes that generate and sustain them. This integrative approach ensures that morphometric analyses capture the complex biological realities underpinning indigenous goat populations.

### Correlation between body weight and linear body measurements

Linear body measurements, body weight and their interrelationships and correlations are important indices determining the genetic potential for improving desired traits during genetic improvement programs. The phenotypic correlation coefficients between body weight and linear body measurements in both males and females are shown in Table 6. In males, highly significant (P<0.001) and positive correlations were found between BW and BL (r=0.84), WH (r=0.83), body depth (RD) (r=0.82), RH (r=0.81), HW (r=0.80) and HG (r=0.80), indicating highly predictable relationships. Other linear body measurements, including the SC, showed a low but positive correlation (r=0.31) with BW. However, the correlation between BW and SH (r=0.68) and EL (r=0.58) were moderate, while HL (r=0.79), DH (r=0.72), RL (r=0.70) and CC (r=0.80) were strongly and positively correlated.

**Table 6:** Correlation coefficients between body weight and linear body measurements (above diagonal for male and below diagonal for female).

	BW	WH	RH	BL	SH	RD	BD	EL	RW	HW	RL	HL	HG	CC	DH	SC
BW	1	0.83⁎⁎	0.81⁎⁎	0.84⁎⁎	0.68⁎⁎	0.82⁎⁎	0.81⁎⁎	0.59⁎⁎	0.58⁎⁎	0.80⁎⁎	0.70⁎⁎	0.79⁎⁎	0.80⁎⁎	0.80⁎⁎	0.72⁎⁎	0.31*
WH	0.62⁎⁎	1	0.98⁎⁎	0.97⁎⁎	0.83⁎⁎	0.89⁎⁎	0.84⁎⁎	0.70⁎⁎	0.71⁎⁎	0.86⁎⁎	0.86⁎⁎	0.93⁎⁎	0.94⁎⁎	0.85⁎⁎	0.85⁎⁎	0.47*
RH	0.58⁎⁎	0.91⁎⁎	1	0.98*	0.85⁎⁎	0.90⁎⁎	0.86⁎⁎	0.70⁎⁎	0.71⁎⁎	0.88⁎⁎	0.88⁎⁎	0.96⁎⁎	0.94⁎⁎	0.86⁎⁎	0.86⁎⁎	0.43*
BL	0.66⁎⁎	0.85⁎⁎	0.87⁎⁎	1	0.81⁎⁎	0.91⁎⁎	0.89⁎⁎	0.72⁎⁎	0.68⁎⁎	0.89⁎⁎	0.88⁎⁎	0.94⁎⁎	0.95⁎⁎	0.87⁎⁎	0.88⁎⁎	0.40*
SH	0.52⁎⁎	0.68⁎⁎	0.70⁎⁎	0.62⁎⁎	1	0.76⁎⁎	0.60⁎⁎	0.69⁎⁎	0.82⁎⁎	0.76⁎⁎	0.83⁎⁎	0.85⁎⁎	0.80⁎⁎	0.71⁎⁎	0.68⁎⁎	0.38*
RD	0.61⁎⁎	0.85⁎⁎	0.84⁎⁎	0.85⁎⁎	0.55⁎⁎	1	0.86⁎⁎	0.59⁎⁎	0.64⁎⁎	0.81⁎⁎	0.74⁎⁎	0.87⁎⁎	0.91⁎⁎	0.79⁎⁎	0.79⁎⁎	0.33*
BD	0.55⁎⁎	0.67⁎⁎	0.68⁎⁎	0.72⁎⁎	0.45⁎⁎	0.73*	1	0.56⁎⁎	0.41⁎⁎	0.82⁎⁎	0.75⁎⁎	0.85⁎⁎	0.88⁎⁎	0.88⁎⁎	0.81⁎⁎	0.28
EL	0.22⁎⁎	0.52⁎⁎	0.55⁎⁎	0.47⁎⁎	0.38⁎⁎	0.44*	0.32*	1	0.48⁎⁎	0.69⁎⁎	0.74⁎⁎	0.68⁎⁎	0.64⁎⁎	0.60⁎⁎	0.53⁎⁎	0.21
RW	0.21⁎⁎	0.21⁎⁎	0.23⁎⁎	0.24⁎⁎	0.22⁎⁎	0.23*	0.19*	0.10	1	0.59⁎⁎	0.67⁎⁎	0.70⁎⁎	0.60⁎⁎	0.55⁎⁎	0.50⁎⁎	0.30*
HW	0.43⁎⁎	0.60⁎⁎	0.61⁎⁎	0.55⁎⁎	0.53⁎⁎	0.48*	0.38*	0.44*	0.23*	1	0.89*	0.91⁎⁎	0.85⁎⁎	0.85⁎⁎	0.80⁎⁎	0.30*
RL	0.46⁎⁎	0.57⁎⁎	0.63⁎⁎	0.63⁎⁎	0.75⁎⁎	0.56*	0.44⁎⁎	0.40⁎⁎	0.25*	0.52*	1	0.90⁎⁎	0.81⁎⁎	0.81⁎⁎	0.78⁎⁎	0.26
HL	0.30⁎⁎	0.52⁎⁎	0.49⁎⁎	0.31⁎⁎	0.66⁎⁎	0.34*	0.18*	0.31*	0.10	0.46⁎⁎	0.40⁎⁎	1	0.91⁎⁎	0.90⁎⁎	0.85⁎⁎	0.36*
HG	0.63⁎⁎	0.78⁎⁎	0.79⁎⁎	0.86⁎⁎	0.48*	0.92*	0.78*	0.41*	0.24*	0.42⁎⁎	0.51⁎⁎	0.16⁎⁎	1	0.86⁎⁎	0.89⁎⁎	0.40*
CC	0.25⁎⁎	0.59⁎⁎	0.51⁎⁎	0.35⁎⁎	0.43*	0.41*	0.23*	0.37*	0.09	0.45⁎⁎	0.24⁎⁎	0.69⁎⁎	0.27⁎⁎	1	0.82⁎⁎	0.27
DH	0.57⁎⁎	0.81⁎⁎	0.80⁎⁎	0.78⁎⁎	0.62*	0.78*	0.55*	0.51*	0.25*	0.61*	0.60*	0.41*	0.74⁎⁎	0.53*	1	0.38*

⁎ P< 0.05

⁎⁎ P<0.01 Body weight: BW; WH: wither height; RH: rump height; BL: body length; SH; sternum height; RD: body depth; BD: bicoastal diameter; EL: ear length; RW: rump width; HW: head width; RL: rump length; HL: head length; HG: hearth girth; CC: cannon bone circumference; DH: muzzle diameter; SC: scrotal circumference

In females, the correlations between BW and BL (r=0.66), HG (r=0.63), WH (r=0.62), RD (0.61), RH (r=0.58), and SH (0.57) were moderate and significant. Other linear body measurements, including the HW (r=0.43), RL (r=0.46), HL (r=0.30), CC (r=0.25), ear length (r=0.22) and RW (0.21), were poorly but positively correlated to BW.

Heart girth consistently emerged as the single best predictor of body weight across both sexes due to its reflection of muscle and fat deposition around the thoracic cavity (Okpeku et al., 2011; Mohammed et al., 2017). This result is in line with findings from Nigerian goats (Okpeku et al., 2011) and Tswana sheep (Bolowe, 2021). The stronger correlations in males suggest more proportional growth patterns, whereas the variability in females likely reflects physiological factors such as gestation and lactation that alter body condition independent of skeletal dimensions.

These correlations have practical implications, as they confirm that simple linear measurements, especially heart girth, can be used as proxies for body weight in field conditions where scales are unavailable. This is particularly valuable for smallholder farmers in resource-limited settings.

### Prediction of body weight from other linear body measurements

Body weight is a crucial growth indicator and an economically significant trait, influencing both market price determination and various routine livestock management practices, such as drug dosage calculations. Consequently, accurately predicting body weight using linear body measurements is essential for effective livestock production (Mohammed et al., 2017).

In this study, stepwise linear regression was employed to develop predictive models for estimating the body weight of male (Table 7) and female (Table 8) Xhosa goats. The regression analysis identified for both sexes. In males, a two-variable model combining heart girth (HG) and body length (BL) explained 73% of the variation in body weight (R² = 0.73). In females, the best model included HG, BL, and sternum height (SH), accounting for 48% of the variation (R² = 0.48). Models using single predictors were less accurate, highlighting the advantage of multivariate approaches.

**Table 7:** Multiple regression analysis of body weight on different linear body measurements in males.

Model	Parameters							
	Inter	*β_1_*	*Β_2_*	R^2^	Adj R^2^	SE	p-value	Std. error of estimate
HG	9.917	0.318	-	0.705	0.702	0.031	<0.001	3.95
HG+BL	7.239	0.182	0.205	0.725	0.719	0.043	<0.001	3.84

HG: Heart girth; BL: body length

**Table 8:** Multiple regression analysis of body weight on different linear body measurements in female.

Model	Parameters								
	Intercept	*β_1_*	*β _2_*	*β_3_*	R^2^	Adj. R^2^	Std. error of the estimates	SE	P-value
HG	-9.459	0.474	-		.441	.439	5.02997	4.21	<0.001
HG+BL	-11.659	0.391	0.191	-	.463	.458	4.94420	3.31	0.001
HG+BL+SH	-10.651	0.219	0.212	0.114	.481	.474	4.87183	2.28	0.025

HG: Heart girth; BL: body length; SH: sternum height

The prominence of heart girth in both models confirms its value as a key indicator of body weight, largely due to its contribution to muscle, fat, and bone components to an animal’s overall body weight (Okpeku et al., 2011). Additionally, heart girth is one of the least affected body measurements by posture and is easy to measure under field conditions, minimizing measurement errors compared to other linear traits (Mohammed et al., 2017). This is consistent with earlier findings in Ethiopian (Asaminew et al., 2016) and Somali goats (Younas et al., 2013). Other studies have also reported the strong correlation between heart girth and body weight, reinforcing its significance as a predictive variable (Asaminew et al., 2016; Asefa et al., 2017; Bolowe, 2021). The inclusion of body length and sternum height in sex-specific models suggests that different body dimensions contribute to body weight prediction depending on physiological differences between sexes. The inclusion of sternum height (SH) in the predictive equation for females is noteworthy. While heart girth and body length captured much of the variation in body weight, SH provided additional explanatory power by reflecting thoracic depth and chest capacity. This measure is particularly relevant in females, where reproductive physiology (gestation and lactation) influences abdominal depth and thoracic shape, leading to variation not fully accounted for by other linear traits.

The predictive regression equations developed were: For males: Y=7.239+0.182HG+0.205BL; Females: Y=−10.651+0.219HG+0.212BL+0.114SH.

These models were selected based on their higher coefficient of determination (R² and adjusted R²) and lower standard error of estimate, indicating strong predictive accuracy. The R² value quantifies the proportion of variability in body weight explained by the model, making these equations valuable tools for field applications where weighing scales may not be available.

Based on the highest Adj R^2^ and lowest std error of the estimate, the stepwise regression equation for predicting the body weight from linear body measurements of male Xhosa goats, therefore, is:

Y(bodyweight)=7.239+0.182HG+0.205BL

The stepwise regression equation for predicting the body weight from linear body measurements of female Xhosa goats, therefore, is:

Y(bodyweight)=−10.651+0.219HG+0.212BL+0.114SH

Adj R^2^ indicates a better fit while adjusting for the number of predictors, while a lower standard error of estimates suggests better prediction accuracy. Importantly, the relatively lower R² in females highlights biological variability, indicating a need for further refinement of models that account for reproductive status or body condition score.

These predictive models are practical tools for smallholder farmers and extension workers in communal farming systems. As weighing scales are often unavailable in rural settings, simple tape-based measurements can serve as effective proxies for body weight estimation. Similar approaches have been successfully applied in Tswana sheep (Bolowe, 2021) and other African small ruminants (Yakubu et al., 2010; Khan et al., 2018). The models developed here, therefore, have direct applications in routine management, drug dosage calculation, and genetic selection.

While this study provides valuable insights, several limitations should be acknowledged. First, the analysis was based solely on phenotypic and morphometric traits without incorporating molecular or genomic data, which could provide deeper insights into genetic diversity and population structure. Second, although efforts were made to standardize measurements, the study relied on field-based data collection under communal farming conditions, where factors such as parasite burden, seasonal forage availability, and variable farmer management may have influenced body measurements. Third, the predictive models, while robust, were developed from goats sampled in three zones of the Eastern Cape and may require validation across other populations and management systems before broader application. Finally, this study did not account for non-genetic factors such as disease incidence or long-term nutritional history, which may also shape body conformation. Recognizing these limitations provides context for the findings and highlights opportunities for future research that integrates genomic tools, longitudinal monitoring, and broader sampling across South African goat ecotypes.

## Conclusion

This study provided a comprehensive phenotypic characterization of indigenous Xhosa goats across three agro-ecological zones in the Eastern Cape Province. Significant morphological variation was observed, with Savanna goats exhibiting superior body traits. Sex and age strongly influenced body weight and linear measurements, as males consistently outperformed females and castrates. Correlation analysis identified heart girth, body length, and body depth as the most reliable predictors of body weight in males, while heart girth, body length, and sternum height were most predictive in females. Stepwise regression models demonstrated strong predictive accuracy, offering practical tools for weight estimation under field conditions where scales are unavailable. These models have direct applications for herd management, selection programs, and genetic conservation.

The study reveals the untapped genetic potential of Xhosa goats for meat production and their adaptability to diverse environments. Our findings also highlight risks posed by uncontrolled crossbreeding and genetic dilution, underscoring the importance of documenting diversity across ecological zones to provide benchmarks for conservation and to inform breeding strategies that enhance productivity while preserving adaptive traits.

This research makes several unique contributions: (i) it delivers the first detailed phenotypic characterization of Xhosa goats across three ecologically distinct zones; (ii) it integrates robust statistical approaches (GLM, correlation, and sex-specific regression models) with assumption testing, ensuring reliable outcomes; (iii) it develops simple, field-applicable models for estimating body weight, addressing a critical gap for smallholder farmers; and (iv) it links phenotypic variation to conservation and breeding implications, establishing benchmarks for sustainable utilization and positioning Xhosa goats as a valuable genetic resource for future improvement programs in South Africa and beyond.

## Funding statement

None.

## Data availability statement

All data generated during this study are included in this publication. Additional datasets used and analysed during the study are available upon reasonable request to the corresponding author.

## Consent for publication

All authors approve the manuscript for publication

## Data and code availability

Data will be provided upon reasonable request

## Declaration of ethical statement

This study received approval for the use of animals based on the guidelines approved by the Animal Research Ethics Committee of the Faculty of Science and Agriculture, University of Fort Hare (Ref: MPE03SNTO01/23/A).

## CRediT authorship contribution statement

**Sibulele Praise Ntonga:** Data curation, Writing – original draft, Investigation. **Oluwakamisi Festus Akinmoladun:** Project administration, Writing – review & editing, Supervision, Methodology, Formal analysis, Conceptualization. **Ziyanda Mpetile:** Methodology, Supervision, Conceptualization.

## Declaration of competing interest

The authors declare that they have no known competing financial interests or personal relationships that could have appeared to influence the work reported in this paper
